# Flexible Electrode Based on PES/GO Mixed Matrix Woven Membrane for Efficient Photoelectrochemical Water Splitting Application

**DOI:** 10.3390/membranes13070653

**Published:** 2023-07-08

**Authors:** Ghadah M. Al-Senani, Mohamed Zayed, Mervat Nasr, Sahar S. Ali, Mohamed Shaban, Fatma Mohamed

**Affiliations:** 1 Department of Chemistry, College of Science, Princess Nourah Bint Abdulrahman University, P.O. Box 84428, Riyadh 11671, Saudi Arabia; 2 Nanophotonics and Applications Lab, Physics Department, Faculty of Science, Beni-Suef University, Beni-Suef 62514, Egypt; 3 Chemistry Department, Faculty of Science, Beni-Suef University, Beni-Suef 62514, Egypt; 4 Chemical Engineering and Pilot-Plant Department, National Research Center, Dokki, Cairo 12622, Egypt; 5 Department of Physics, Faculty of Science, Islamic University of Madinah, P.O. Box 170, Madinah 42351, Saudi Arabia; 6 Materials Science Research Laboratory, Chemistry Department, Faculty of Science, Beni-Suef University, Beni-Suef 62514, Egypt

**Keywords:** photoelectrochemical H_2_ production, conversion efficiency, polyether sulphone (PES), mixed matrix woven membranes, hummer’s method, phase inversion method

## Abstract

We introduced, for the first time, a membrane composed of nanostructured self-polyether sulphone (PES) filled with graphene oxide (GO) applied to photoelectrochemical (PEC) water splitting. This membrane was fabricated through the phase inversion method. A variety of characteristics analysis of GO and its composite with PES including FTIR, XRD, SEM, and optical properties was studied. Its morphology was completely modified from macro voids for bare PES into uniform layers with a random distribution of GO structure which facilitated the movement of electrons between these layers for hydrogen production. The composite membrane photocathode brought a distinct photocurrent generation (5.7 mA/cm^2^ at 1.6 V vs. RHE). The optimized GO ratio in the membrane was investigated to be PG2 (0.008 wt.% GO). The conversion efficiencies of PEC were assessed for this membrane. Its incident photon-to-current efficiency (IPCE) was calculated to be 14.4% at λ = 390 nm beside the applied bias photon-to-current conversion efficiency (ABPE) that was estimated to be 7.1% at −0.4 V vs. RHE. The stability of the PG2 membrane after six cycles was attributed to high thermal and mechanical stability and excellent ionic conductivity. The number of hydrogen moles was calculated quantitively to be 0.7 mmol h^−1^ cm^−2^. Finally, we designed an effective cost membrane with high performance for hydrogen generation.

## 1. Introduction

Utilizing renewable energy sources, such as solar energy to produce electricity or hydrogen, has recently become a popular topic. One promising method for removing pollutants from sewage is photoelectrochemical (PEC) technology, which can also split water to produce an H_2_ fuel [1]. The type of semiconductor catalysts, as well as their inherent photoelectrochemical and photocatalytic capabilities, have a considerable impact on PECs performance in the hydrogen generation process [2]. Therefore, new catalysts are needed for this purpose. Due to its inherent qualities, including its high proton conductivity and favorable mechanical and thermal properties, Nafion is used as an appropriate electrolyte membrane in the production of hydrogen [3,4]. Although it has outstanding qualities, high cost, and its potential applications have poor proton ion selectivity [5]. Due to its distinct qualities, such as high thermal stability, low cost, and high chemical resistance, the polyether sulfone (PES) membrane is a prospective replacement membrane for Nafion in energy applications. PES has a high-water uptake capacity but also low stability and a low retention capacity at higher temperatures, which reduces its proton conductivity [6]. Incorporating nanoparticles [7,8] crosslinking [9,10], and ionic liquid composites [11,12] are a few methods that have been used to increase the material’s ability to retain water for high-temperature applications.

Graphene oxide (GO) is produced when graphite is oxidized because numerous oxygen functional groups establish bonds with the carbon plane [13]. The behavior of GO is usually a p-type semiconducting [13]. GO is hydrophilic as well, making it simple to spread in aqueous solutions. For enhancing conductivity and water retention in the membrane matrix, graphene oxide (GO) has been regarded as an alluring organic filler [14]. Due to its affordable material, desired photo corrosion resistance, and low price, it offers important features. Additionally, it has a great ability to transmit ions and electrons between various devices [8,15]. Due to its ability to transport electrons, GO was employed by Kusuma et al. [16] in quantum dots-sensitized solar cells (QDSSCs). Perovskite/GO photocatalyst for CO_2_ reduction was studied by Xu et al. [17]. The addition of sulfonated GO to PES is anticipated to improve the proton exchange membrane (PEM) mechanical and water-retention properties as well as its proton conductivity [18]. GO is considered a promising candidate in hydrogen cells due to the richness of their surface with hydroxyl, epoxy, and carboxyl groups which are responsible for the promotion of the proton exchange through hydrogen-bonding networks [19]. Many researchers studied the role of the aromatic polymer matrix including GO or its derivatives on the proton conductivity and the permeability to the reactant [20]. Poly(2,5-benzimidazole)-grafted GO was impregnated by Ko et al. [21] into the sulfonated poly(arylene ether sulfone) SPAES matrix. In comparison to virgin SPAES, the composite membranes demonstrated superior proton conductivity and dimensional stability. The mechanical quality and oxidative stability of the composite membrane were improved, but the proton conductivity was decreased, according to Zhao et al. [22], who also described a series of unique ionic cross-linked sulfonated poly(ether sulfone) composed with quaternized GO. A high proton conductivity was also demonstrated by the SPAES membranes paired with GO derivatives, however, this resulted in a high swelling ratio [23,24].

Composite membranes made of PES and nanostructures have good thermal, mechanical, and oxidative characteristics. The phase inversion method is the most popular approach for PES-based membrane synthesis because of its simplicity, low cost, and high output. This is because the hydrophobicity of the PES composite membrane plays a key role. This method is also more practicable than previous approaches for enhancing the performance of polymer matrices with different nanostructures [25]. To increase the effectiveness of PEC H_2_ generation, this work intends to produce a novel membrane that is both affordable and based on PES and GO nanosheets. The number of hydrogen moles will also be calculated, and other necessary characteristics of this membrane will be studied. All PEC tests, including the current density-potential (J-V) under white light and monochromatic illuminations and current density-time (I-t) tests, were assessed. The conversion efficiencies of the PEC process are also evaluated. 

Our work’s PES/GO nanosheets with varying GO weight% ratios properly retain the advantages of graphene oxide and polyether sulphone to develop new, affordable, and flexible membranes for PEC hydrogen generation. This enables us to achieve high PEC efficiency and performance, i.e., the addition of GO with PES can enhance the generation of H_2_ and improve the stability of the electrode. The main purposes are to (1) examine the optimum added ratio of GO nanostructures for designing the H_2_ production system, and (2) estimate the number of hydrogen moles, as well as other necessary characteristics of the studied membrane. All PEC tests were assessed, including the current density-potential (J-V) under white light and monochromatic illuminations and current density-time (J-t) tests. The conversion efficiencies of the PEC process are also evaluated.

## 2. Experimental Details

### 2.1. Raw Materials

Polyether sulfone (Ultrason E6020P, JIAHUA CHEMICALS INC, Shanghai, China), N,N-dimethylformamide (DMF) (CAS No. 68-12-2, BASF Company, Schwarz Heide, Germany), Polyethylene glycol (PEG), H_2_SO_4_, and H_3_PO_4_ were purchased from Sigma-Aldrich, Schnell Dorf, Germany. The graphite was purchased from Laborchemie, Apolda, Apolda, Germany, and H_2_O_2_ and KMnO_4_ were obtained from the Egyptian Adwic Company, Cairo, Egypt.

### 2.2. Synthesis of GO Nanosheets

By using a modified Hummer method, GO nanosheets have been manufactured. Either graphite flakes or natural graphite powder can be used as a starting material for the manufacture of GO. Graphite flakes were used as a starting material for GO fabrication due to their advantages such as low price, high electron conductivity, and high yield [26]. In addition, larger GO sheets may be produced at a lower price if graphite flakes are used as the starting material. Conversely, finer graphite might result in a lower yield and a higher degree of oxidation. As follows, we were able to make bigger GO sheets from graphite flakes with a high yield and a low cost for use in PES/GO membranes. Briefly, under a magnetic stirrer (350 rpm), 1 g of graphite flakes was suspended in a mixture of 120 mL of sulfuric acid and 14 mL of phosphoric acid. Subsequently, 6 g of potassium permanganate was gradually added within 1 h in an ice bath. Then, the final obtained solution remained at a low temperature (50 °C) for one day. The next stage was to reduce the KMnO_4_ residual and generate a considerable amount of air bubbles by adding 10 mL of H_2_O_2_ and 800 mL of deionized H_2_O (DW) drop by drop alternately to the suspended graphite solution in an ice bath. After finishing this procedure, we see that the solution’s hue has changed to radish yellow. When the pH of the solution reached nearly 7, the precipitated powder was collected and repeatedly washed with DW and methanol. The neutral solution was subsequently dried using a muffle furnace (60 °C) to deliver GO nanosheets as a powder.

### 2.3. Fabrication of PES/GO Mixed Matrix Woven Membranes

PES/GO mixed matrix woven (MMW) membranes were prepared by using the phase inversion method to further use in Photoelectrochemical water splitting applications. The casting solutions of PES were formulated by dissolving different weight ratios (20 wt.% PES, 1 wt.% PEG) in DMF (as a solvent) with different weight ratios of GO (0, 0.004, 0.008, 0.01, 0.08, and 0.1 wt.%). The mixture was stirred for one day at nearly 55 °C to obtain a homogenous solution. GO nanosheets with different weight ratio was dispersed in casting PES solution via an ultrasonic device (for 5 h at 25 °C) to obtain a regular dispersion of GO and remove air bubbles. Using a casting knife and a coated woven fabric support material, a polymeric membrane solution was cast with a total measured casting thickness of 215 μm before being swiftly submerged in water as a non-solvent bath. The manufactured membranes were left in tanks of fresh DW for one day, or until the phase inversion process was confirmed to be complete and the membrane had separated from the glass plate. The membrane was peeled and then cleaned with DW to get rid of any remaining solvent. The produced membrane was then employed for additional characterization after being kept in fresh DW. The obtained samples are labelled PG0, PG1, PG2, PG3, PG4, and PG5 according to loaded wt.% GO into PES of 0, 0.004, 0.008, 0.01, 0.08, and 0.1, respectively.

### 2.4. Samples Characterizations

X-ray diffraction technique (XRD; PANalytical X’Pert Pro, Amsterdam, The Netherlands) with a copper source (CuK_α1_, λ = 1.5406 Å) was used to investigate the structural properties of fabricated GO nanopowder with a step size of 0.04°. A field emission scanning electron microscopy unit (ZEISS Sigma 500 VP FE-SEM) was used to study the top, bottom, and cross-section morphologies for the fabricated samples. An energy-dispersive X-ray analysis (EDAX; Oxford Link ISIS 300, Concord, MA, USA) was used to study the chemical composition and the ratio of the synthesized GO. A Raman spectrometer (Thermo Fisher Scientific, model DXR3xi, Waltham, MA, USA) with a 532 nm laser was used to check the purity of the GO powder. Fourier transform infrared spectroscopy (ATR-FTIR, VERTEX 70, Bruker, Germany) in the range of 600–4000 cm^−1^ was used to recognize the functional groups of the GO and prepared membranes. The optical properties were examined by using a double-beam spectrophotometer (PerkinElmer, Lamba 950, Waltham, MA 02451, United States, USA) to study the optical properties of fabricated PES and PES/GO electrodes.

### 2.5. Photoelectrochemical (PEC) Water Splitting Measurements

The PEC measurements were conducted by using an OrigaFlex electrochemical workstation (OGFEIS connected to an OGF500 Pack) in conjunction with a three-electrode configuration. The working, reference, and counter electrodes employed were the photocathode (PEC/GO), a silver-silver chloride (Ag/AgCl), and a platinum electrode. Similar configurations were reported by Joy et al. [27] and Sekizawa et al. [28]. The silver paste was coated on one edge of the working electrode to enhance the ohmic electrical contact. Next, 0.3 M of sodium sulfate (Na_2_SO_4_) aqueous solution with a neutral medium (pH 7) was used as an electrolyte to utilize the photo-generated holes from the photocatalytic surface. The photocurrent density responses were recorded using the electrochemical workstation in the linear sweep voltammetry (LSV) mode by using a 400-Watt Xenon lamp (Newport, 66142-500HX-R07, Newport, UK) providing conventional white light illumination equivalent to one sun (AM 1.5; 100 mW cm^−2^). The light source is provided with a set of bandpass filters with wavelengths ranging from 390 to 636 nm to obtain monochromatic lights. The applied scanning voltage (V) ranged from −1 to +1 V. Note that, the data were collected in triplicates, and the average values are presented. For electronic conductivity, the (I-V) characteristic curves for all PEC/GO electrodes were measured at room temperature under applied voltage (V) varying from 0 to 1 V with a step of 0.1 V via a two-electrode system. The silver paste was used to provide the Ohmic contact, and the curves were measured using the electrochemical workstation. The ionic conductivity for all PEC/GO electrodes was examined through (I-V) characteristic curves using 0.3 M sodium sulphate (Na_2_SO_4_) aqueous solution with a neutral medium (pH 7) under applied voltage (V) varying from −0.5 V to 0.5 V via the three-electrode system.

## 3. Results and Discussion

### 3.1. Characterization of Fabricated GO Nanosheets

**Figure 1:**
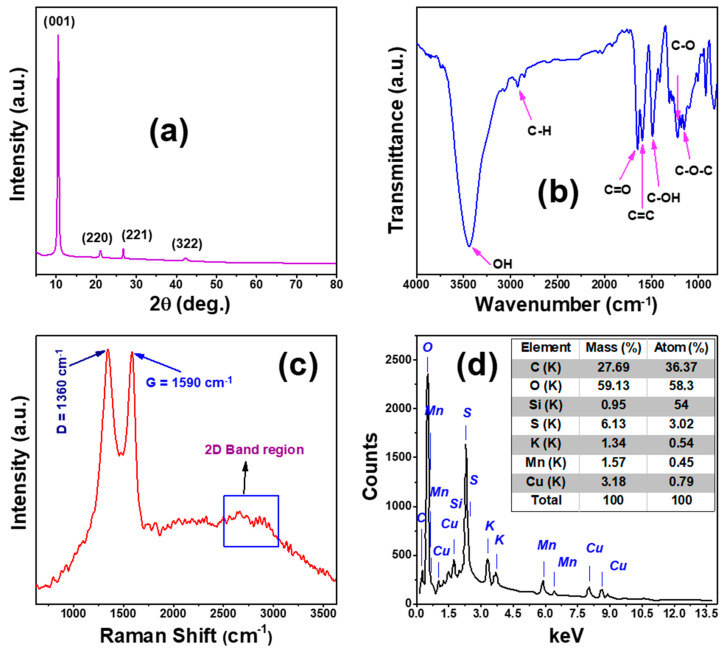
(**a**) XRD chart, (**b**) FTIR spectrum, (**c**) Raman analysis, and (**d**) EDX chart of GO nanosheets.

The XRD chart, FTIR spectrum, Raman analysis, and EDX chart of GO nanosheets fabricated by a modified Hummer method are illustrated in Figure 1.

#### 3.1.1. XRD of GO Nanosheets

Figure 1a shows the crystallographic structure of the manufactured GO nanosheets. GO showed a tetragonal polycrystalline wurtzite structure with space group (P 42/n) according to matching card JCPDS No. 96-590-0025 [29]. The production of GO was confirmed by a sharp characteristic XRD peak positioned at 2θ = 10.50° according to crystal plane (001), which is attributed to the interlayer properties of the GO nanosheets [12]. In addition, there are other three minor peaks for GO located at 2θ = 21.05°, 26.73°, and 42.26° assigned to hkl planes (220), (221), and (322), respectively. No additional peaks of crystalline impurities or metallic GO phases are detected on the XRD graph, suggesting the creation of a highly pure crystalline sample of GO nanosheets by modified Hummer’s method.

The subsequent interlayer spacing (d-spacing) of the fabricated GO nanosheets was estimated and listed in Table 1 by using the Bragg equation [29]:

(1) nλ=2 d sin sin θ

where n is the order of diffraction, λ is the wavelength of the X-ray (1.5406 Å), and *θ* is Bragg’s diffractometer angle. From Table 1, the d-spacing of the (001) plane is 8.423 Å. The starting interlayer spacing of the graphite (G) is nearly 3.4 Å, which indicates that all prepared GO is oxidized and the exfoliation process as the presence of oxygen functional groups increases the distance between the layers in the G structure [30].

The crystallite size (*D*) of GO nanosheets was assessed and listed in Table 1 by using the obtained values of the peak full width at half maximum (*β*) from XRD analysis by Scherrer’s equation [31]:

(2) D=(0.94 λ)/β cos θ

From Table 1, the average crystallite sizes for GO are 23.17, 19.47, 40.49, and 10.19 nm for (001), (220), (221), and (322), respectively.

To recognize the preferred orientation for GO growth, the texture coefficient (*TC* (*hkl*)) was calculated by the following equation [32]:

(3) $$TC\left(hkl\right)=\frac{I_{rel.}}{\left(N^{-1}\right)*(\sum I_{rel.})}$$

where *I_rel._* is the relative intensity (ratio between measured intensity (*I*) and the standard intensity (*I_o_*) obtained from the GO card), and *N* is the number of diffraction peaks. The maximum intensity, (001), is found at 2 θ = 10.50° and has a TC of 3.66, which indicates that it is growing in the preferred orientation, according to TC calculations (Table 1) and the XRD chart in Figure 1a. (220), (221), and (322), respectively, have TC values of 0.11, 0.18, and 0.05.

The lattice parameters (*a* = *b*, and *c*), the unit cell volume (*V*), and dislocation density (*δ*) of the manufactured tetragonal GO powder are calculated by using the following equations [33,34]:

(4) $$\frac{1}{d^{2}(hkl)}=\frac{\left(h^{2}+k^{2}\right)}{a^{2}}+\frac{l^{2}}{c^{2}}$$

(5) $$V=a^{2}\;*\;c$$

(6) $$\delta =\frac{1}{D^{2}}$$

From Table 1, the lattice parameters (*a*, *c*) and *V* are 11.94 Å, 4.33 Å, and 617.3 Å^3^, respectively, which is nearly equal to standard card data values. The dislocation densities for the fabricated GO are 1.9 × 10^−3^, 2.6 × 10^−3^, 61.1 × 10^−3^, and 9.6 × 10^−3^ dislocation/nm^2^ for (001), (220), (221), and (322), respectively. The low values of GO dislocation densities indicate the decline in the number of defects and impurities, which confirms the purity of fabricated GO by the modified Hummer method.

#### 3.1.2. FT-IR Analysis of GO Nanosheets

After the GO nanosheets were completely collected and dried, the FTIR evaluation was performed. FT-IR is a destructive analysis technique used to identify the chemical interactions and the bonds between functional groups of GO nanosheets. Figure 1b shows the FT-IR chart for the manufactured GO nanosheets. From this figure, there is a sharp intense peak around 3444 cm^−1^ belonging to stretching vibrations of a hydroxyl group (O-H), which is related to the absorbed H_2_O molecules in GO [35,36]. There are additional peaks with low intensities located at wave numbers 2925, 1650, 1599, 1490, 1220, and 1153 cm^−1^ that are related to the stretching band sp3 (C-H), stretching vibration bond of the carbonyl groups (or carboxylic groups) (C=O), vibration band as a part of the phenol ring in GO structure (C=C), stretching vibration of alcohol (C-OH), stretching bond of an epoxy group (or carboxylic acid) (C-O), and (C-O-C) due to stretching vibration of GO, respectively. These results are similar to that observed in previous research on GO [37,38]. FT-IR analysis confirms the creation of a pure crystalline GO, as indicated in the XRD analysis (Figure 1a). The presence of oxygen groups reveals that the graphite powder has been completely oxidized. The polar groups, exclusively hydroxyl groups, resulting in the growth of hydrogen bonds between graphite and molecules of water; this further supports the hydrophilic nature of GO.

#### 3.1.3. Raman Analysis for GO Nanosheets

The Raman spectrum of the GO nanosheets is presented in Figure 1c. The three major peaks in the Raman spectrum of GO are the D peak, G peak, and 2D band, which are sited at 1343, 1585, and 2655 cm^−1^, respectively [39]. The D band is due to the breathing mode of k-point phonons of A_1g_ symmetry of the defects in the sp^3^ hybridized carbon bonds, the G band is assigned to the first order scattering of the E_2g_ phonons of the sp^2^ hybridized carbon atoms, and the 2D band is due to stacking of GO sheets [40].

#### 3.1.4. EDAX of GO Nanosheets

The energy dispersive X-ray analysis (EDAX) was performed for GO at a utilized voltage of 15 kV to recognize the chemical composition and its elemental ratios. The EDAX is captured by the Si detector and plotted in Figure 1d with an inset table that gives the quantitative analysis of GO nanosheets’ chemical composition ratios. According to Figure 1d and the inset Table, there is an alone peak for O located at 0.53 keV, and the other peak associated with C at 0.28 keV. The high atomic ratios of C and O (36.37%, and 58.30%) are owing to the GO structure. This proves that GO with high purity was formed. These results are consistent with XRD analysis. However, there are additional minor elements such as Si, Mn, S, Cu, and K. The main reason for the presence of Mn, K, and S in the EDX chart is owing to the use of KMnO_4_ and H_2_SO_4_ as oxidizing agents during the fabrication of GO by the modified Hummer method. While Cu and Si appear due to the chamber effect of SEM-EDX not due to the graphene oxide content [41].

#### 3.1.5. STEM Analysis of GO

**Figure 2:**
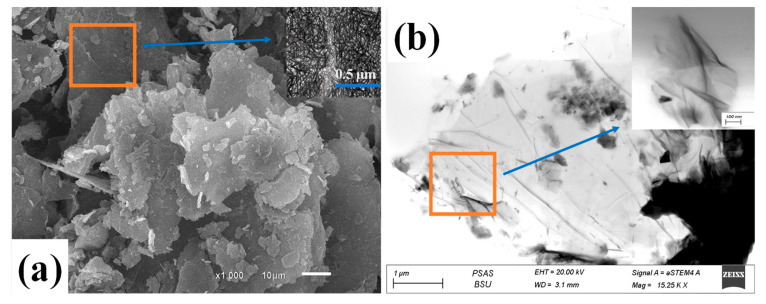
(**a**) SEM and (**b**) STEM images of GO nanosheets.

Figure 2 illustrates the top surface morphology of typical GO nanosheets by using SEM and STEM. From Figure 2a, the image shows a uniform layer with a random distribution of cracked nanosheets of GO structure with small wrinkles. In addition, the inset magnified image of Figure 2a illustrates that the GO NS is composed of a porous-like structure, which forms similar to a network structure. Azam Marjani et al. observed similar results when fabricating GO by the modified Hummer method [39].

From Figure 2b, the STEM analysis displayed a GO structure of stacked nanosheets with wrinkled edges. Furthermore, regular nanosheets with cracked structures happened during the oxidation process during the fabrication method. Furthermore, from the magnified image of Figure 2b, we notice that GO folded nanosheets of a particular surface area. This may be due to van der Waals’s interaction between every two close GO nanosheets. The agglomerations of GO nanosheets occurred during the drying step after complete fabrication due to the mesoporous-like structure with the enhanced surface area of GO [41].

### 3.2. Characterization of PES/GO MMW Membranes

#### 3.2.1. FT-IR Analysis of PES/GO MMW Membranes

**Figure 3:**
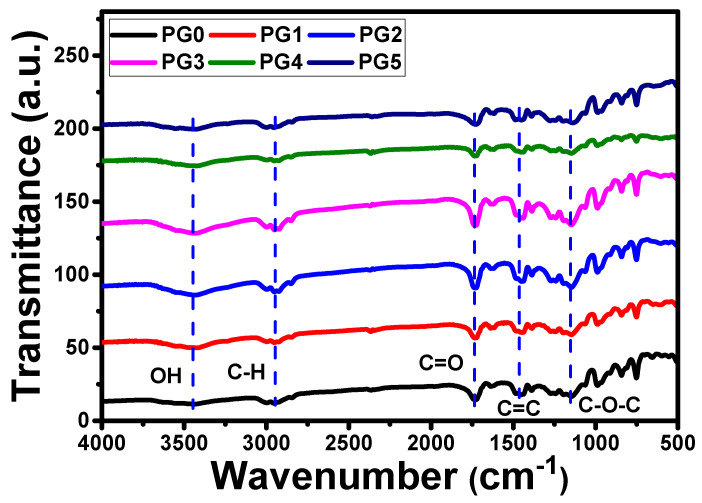
FT-IR spectra of bare PES and PES/GO MMW membranes with different GO nanosheets ratios.

The FT-IR investigation was performed after the preparation of bare PES and PES/GO MMW membranes with different GO nanosheets weight ratios. The FT-IR study was used to identify the chemical compositions and the functional groups of the prepared membranes as illustrated in Figure 3. From Figure 3, the peak intensity of hydroxyl groups located at 3100–3600 cm^−1^ increased as the GO nanosheets wt.% content loaded increased. Additionally, the C=O peak intensity increased with increasing the GO wt.% content till PG3. For GO wt.% content >0.01 wt.%, the intensities of these peaks decrease. The presence of boarding peaks confirmed the successive incorporation of GO nanosheets in the PES matrix. There are other minor peaks detected in the IR spectrum related to PES. The peak at around 1295 cm^−1^ is arising due to O=S=O asymmetric stretching, whereas the symmetric stretching of O-S-O gives a band at around 1150 cm^−1^ [42,43].

#### 3.2.2. Surface Morphology of PES/GO MMW Membranes

**Figure 4:**
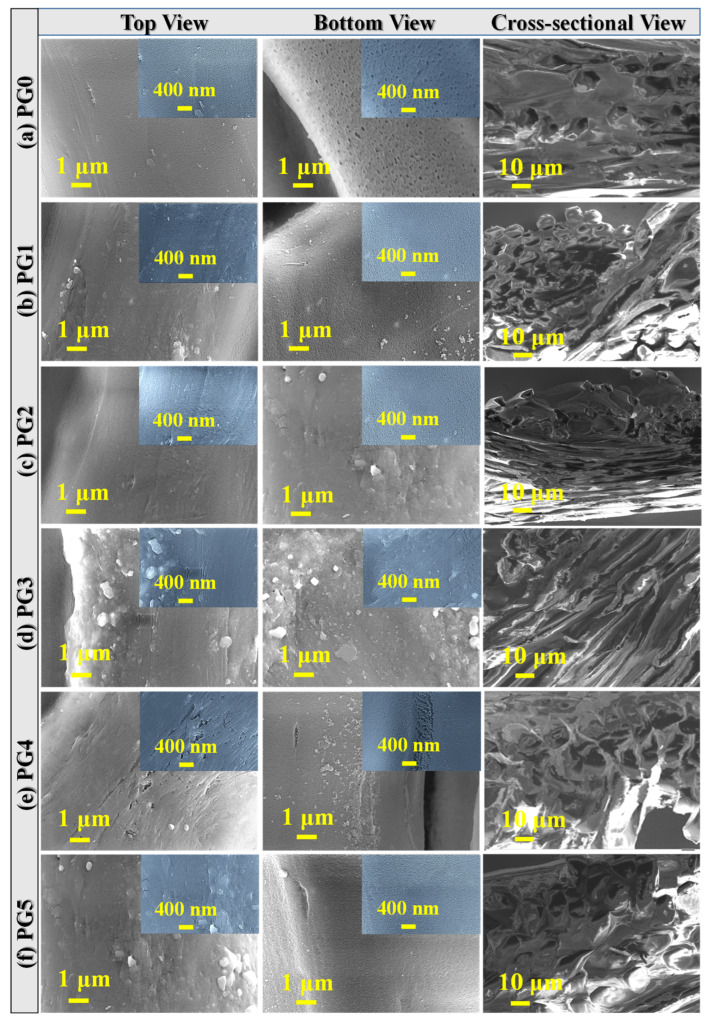
SEM images of top views, bottom views, and cross-sectional views for PES/GO MMW membranes with different GO wt.% ratios.

The SEM images of top surfaces, bottom surfaces, and cross-sections of bare PES and PES/GO MMW membranes with different GO nanosheets ratios are displayed in Figure 4a–f. From Figure 4, there is a significant variation between the surface images of the bare PES and mixed PES/GO MMW membranes. The FE-SEM images proved the distribution of GO nanosheets through the top/bottom surfaces of PES membranes. This is due to the hydrophilic nature of GO nanosheets and their good dispersion in the polymer matrix (PES). Furthermore, because of the hydrophilic nature of GO nanosheets, a rapid exchange of solvent and non-solvent occurs during the phase inversion process, resulting in increased porosity and alterations in the macro-void structure [44]. Figure 4e,f shows GO nanosheet aggregation in the membrane surface at higher GO wt.% inclusion ratios (PG4 and PG5). This could be because GO nanosheets have a carbon-based structure. The cross-section images of the manufactured membranes (Figure 4) revealed that the integrated GO ratio had a significant impact on the morphology of the membrane.

The cross-section of bare PES, Figure 4a, shows its macro-void structure, which is drastically altered by the insertion of GO nanosheets into the polymer matrix during the phase inversion process. When the GO nanosheets wt.% ratio was increased to 0.01 wt.% (PG3), the macro-voids expanded from the top to the bottom surface, as illustrated in Figure 4d. By raising the GO nanosheets content over 0.01 wt.%, the GO nanosheets agglomerated and macro voids are transformed into layer structure, as shown in Figure 4f at PG5. Double skin-layer structures were observed after raising the GO nanosheets loading ratio to 0.1 wt.% as shown in Figure 4f. In common, bare PES and all PES/GO MMW membranes had an irregular nanoscale porous sub-layer structure with a thick top layer and porous layers at the middle and bottom. Furthermore, the pore size reduces from bare PES membrane to GO 0.01 wt.% ratio (PG3), then increases with increasing GO nanosheets at PG4 and PG5 samples.

#### 3.2.3. Optical Properties of PES/GO MMW Membranes

**Figure 5:**
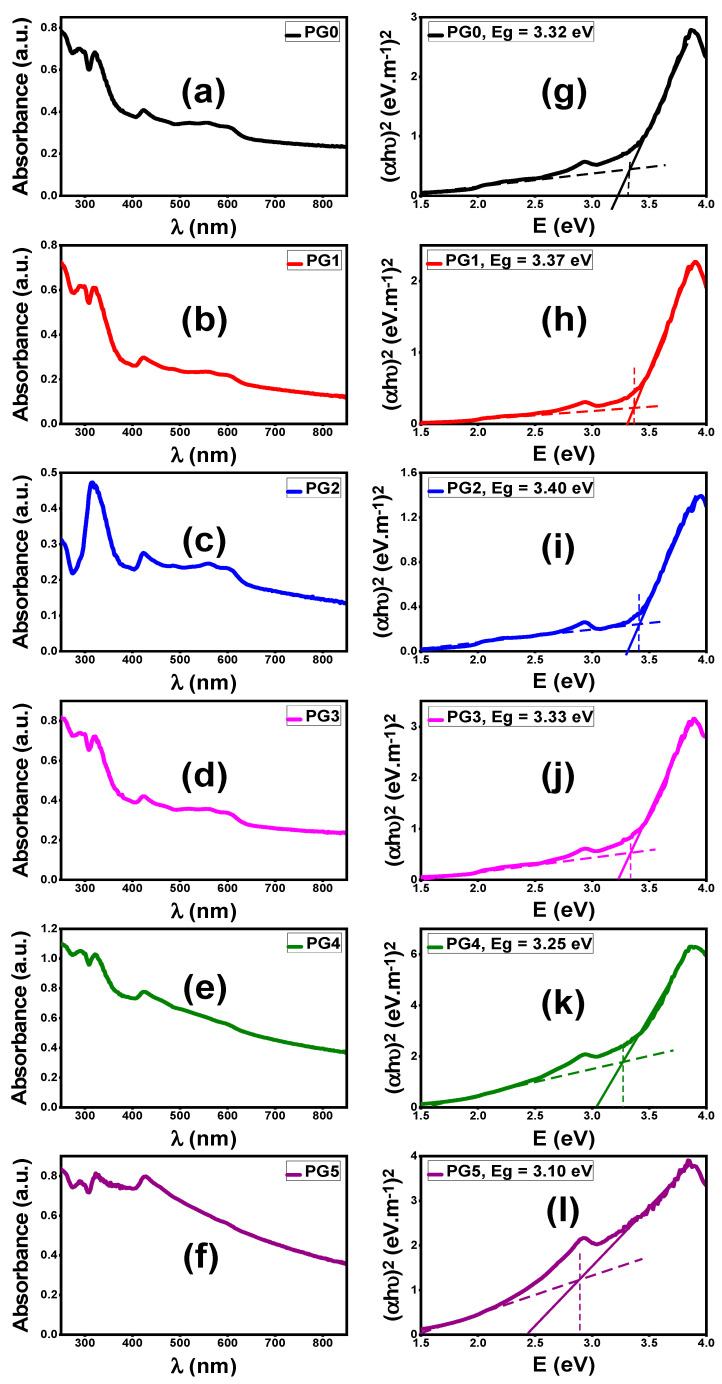
Optical properties of bare PES and PES/GO MMW membranes with different GO nanosheets wt.% ratios; (**a**–**f**) absorbance spectra and (**g**–**l**) (αhυ)^2^ versus E = hυ.

Optical spectroscopy is a strong method to explore the optical analysis and energy bandgap of nanostructured materials in the UV–visible region (250–850 nm). The absorbance (A%) spectra of PES and PES incorporated by GO nanosheets with different weight ratios are displayed in Figure 5a–f. The glass substrate was used as a reference for the measurements of manufactured membrane absorption; thus, background subtraction was applied to the absorption spectra in Figure 5a–f. All PES/GO MMW membranes show two strong absorption bands in the UV/visible region (around 321 and 427 nm) resulting in electrons jumping from the valence band to the conduction band of PES/GO. This can result in high photocatalytic activity in the UV/visible region of the PES/GO MMW membranes. The absorbance values are significantly affected by the incorporation of GO into the PES matrix. For comparison, at λ = 321 nm, the absorbance value decreases from 0.67 @ pristine PES (PG0) to 0.47 @ PG2. This could be ascribed to the loaded GO nanosheets being well disseminated in the polymer matrix (PES), as shown in SEM images of Figure 4b,c. While the absorbance increased for PG4 and PG5 at the same wavelength (321 nm). This may be attributed to the formation of double skin-layer structures (GO on the top and bottom surfaces of PES), as demonstrated in the SEM image in Figure 4d,e.

For the direct bandgap transition of electrons from the VB to CB, the energy bandgap can be estimated by using the Tauc equation [31]:

(7) $${\left(\alpha \;E_{ph}\right)}^{2}=X\;(E_{ph}-E_{g})$$

where α, *E_ph_*, *E_g_*, and X are absorption coefficients, the energy of the incident photon, the energy bandgap, and the independent energy constant, respectively. The bandgap (*E_g_*) values were estimated according to the intersection between the pre-edge extended line and the major linear tangent (dashed lines, Figure 5). From Figure 5g–l, the *E_g_* value of PG0 was 3.32 eV. With the low content of loaded GO nanosheets, the *E_g_* increased to 3.40 eV for PG2. After that, by increasing the loaded GO nanosheets to PG5 the *E_g_* decreased to 2.90 eV. The dramatic change in the values of the optical band gap is related to the changes in the composition and membrane nanomorphology. The optical band gap is decreased when the GO content rises as a result of the formation of localized states at the band gap’s edges. In addition, further reduction in the optical band gap is ascribed to the aggregation of GO on the top and bottom surfaces of PG4 and PG5 membranes (Figure 4), which occurs at high GO concentrations. While a low concentration of localized states led to a widening of the optical band gap, as evidenced by the shifting of the absorption band’s edge to a lower wavelength as shown for PG1 and PG2 (Figure 5b,c). This value is appropriate for use in the photoelectrochemical (PEC) catalytic generation of solar hydrogen.

## 4. PEC H_2_ Generation

### PEC Characteristics and Conversion Efficiencies

**Figure 6:**
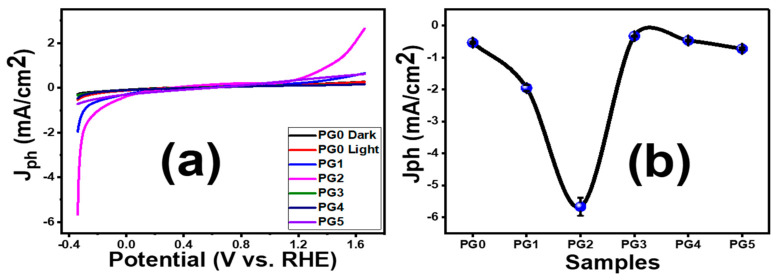
(**a**) Effect of light on the photocurrent density-potential (J_ph_-V_RHE_) curves, and (**b**) effect of different ratios of GO on the produced J_ph_ by the prepared membranes. Error bars are provided in (**b**) for triplicate measurements.

In photoelectrochemical (PEC) measurements, three-electrode systems were used and 0.3 M Na_2_SO_4_ (0.3 mol/L) solution was employed as the electrolyte. The photocurrent change with applied voltage was recorded using the OrigaFlex potentiostat. Figure 6a shows the results of PEC experiments conducted on PES membranes with GO wt.% ranging from 0 to 0.1 in both the presence and absence of white light irradiation. In the dark, the current density is at its lowest value, 0.28 mA/cm^2^ for PG0. However, there was a considerable improvement in current density under white light illumination. This increased photocurrent density was attributed to the photocathode materials’ considerable solar spectrum sensitivity. The observed photocurrent shows that the planned membranes are highly photoactive. Figure 6b also demonstrates that the PEC response is affected by the incorporated GO ratio in the membrane. The incorporation of GO into the membrane matrix speeds up electron transfer and reduces the e^−^/h^+^ recombination. The PG2 photocathode has the maximum photocurrent density (5.7 mA/cm^2^), which is 10.1 times greater than the photocurrent of bare PES (0.5 mA/cm^2^). The PG2 membrane showed the greatest light response of all the examined GO-doped photoelectrodes. In general, the loaded GO nanosheet enhances conductivity and inhibits photocorrosion of photoelectrodes [45]. The increase was attributed to PESs good interaction with GO via the van der Waals force [46]. The optimal amount of GO is found in the PG2 membrane. This also agrees with the measured current-voltage characteristics for the membranes as shown in Figure S1a (Supplementary data). From these curves, the electric conductance of the membranes is estimated and presented in Table S1 (Supplementary Data). The highest conductance is estimated for the PG2 to be 0.09717 ± 4.57686 × 10^−4^ μS for a sample with an area of 1 cm^2^. A slight reduction in conductivity was observed with a further increase in the GO content, which can be attributed to GO nanosheets aggregating on the membrane surface at higher GO wt.% as shown in the SEM images of Figure 4 for PG4 and PG5. The ionic conductivity shows similar behaviors as shown in Figure S1b and Table S1, whereas PG2 showed ionic conductivity of 2.71506 ± 0.00307 mS versus 0.10096 ± 0.00166 mS for PG5. I.e., the improvement of membrane performance can be correlated with the improvement of ion/electric conductivity. Similarly, Yang et al. [47] found that increasing the electrical conductivity of electrodes enhanced the amount of catalyst and reaction sites utilized for the oxygen evolution reaction. The enhancement was attributed to the electron pathway’s involvement in dimensionally expanding the water-splitting reaction sites in liquid electrolytes, improving the reaction kinetics, and lowering cell ohmic resistance. Additionally, according to Du et al. [48], organic/inorganic composites have been proposed as a way to enhance the anion-exchange performance and chemical and mechanical stability of anion-exchange membranes for water splitting. Ions are transported by the electrolyte’s ionic conductivity to complete the redox reaction to maintain a reasonable power output, which improves the efficiency of hydrogen production [49]. Note that the excess GO may hinder light absorption, resulting in a decrease in photocurrent density. This scenario reduces the number of electrons excited from the valence band to the membrane’s conduction band [50].

**Figure 7:**
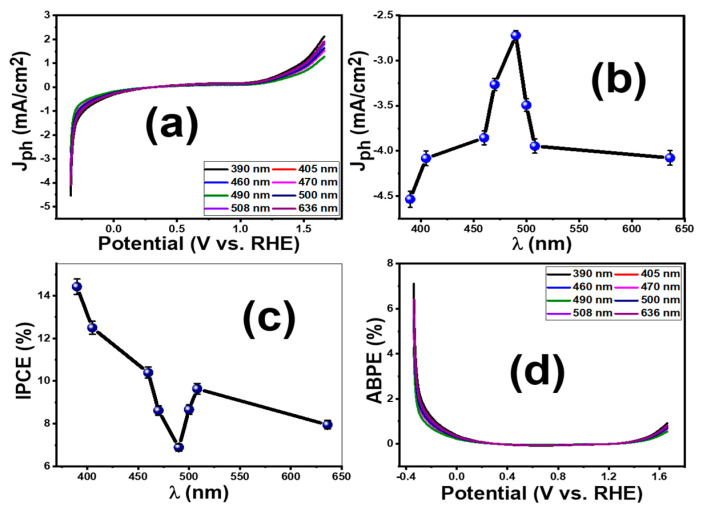
(**a**) Effect of monochromatic wavelength on J_ph_-V curves, (**b**) J_ph_ versus the wavelength, (**c**) IPCE (%) versus the wavelength, and (**d**) ABPE (%) versus the applied potential for the optimum PG2 membrane. Error bars are provided in (**b**,**c**) for triplicate measurements.

The photoresponse of PG2 under monochromatic illumination utilizing optical filters of different wavelengths (390–636 nm) was studied and shown in Figure 7a. The value of photocurrent density (J_ph_) changed dramatically when the monochromatic light wavelength changed. According to Figure 7b, the PG2 photoelectrode can absorb the visible range of sunlight and produce the maximum photocurrent density [51]. The maximum J_ph_ was 5.7 mA/cm^2^ at 390 nm.

The applied bias photon-to-current conversion efficiency (ABPE) and incident photon-to-current efficiency (IPCE) calculations were used to evaluate the conversion efficiencies of the optimized PG2 membrane [52,53]. The values of IPCE are calculated using Equation (8) [52].

(8) $$IPCE(\%)=\frac{{1240\times J}_{ph}(mA/{cm}^{2})}{\lambda \left(nm\right)\times P(mW/{cm}^{2})}\times 100$$

where $J_{ph}$ (mA/cm^2^) is photocurrent taken at a certain wavelength for incident photon; *λ* is the wavelength of the monochromatic light, and $P(mW/{cm}^{2})$ is the illuminating light power density. The values of the incident photon-to-current efficiency (IPCE) were assessed from 390 to 636 nm at −1 V and presented in Figure 7c. The maximum IPCE value for the PG2 photoelectrode was calculated to be 14.4% at *λ* = 390 nm. Its significant increase in the IPCE value could be ascribed to the incorporation of GO over PES which facilitates the charge separation and transfer. Due to its high electron mobility, GO has a good ability to capture electrons in electrochemical cells and a rapid transfer of charge, reducing the risks of recombination [54]. Notably, all IPCE values in the visible range are the highest when compared to all regions of sunlight, which is due to the similarity of their optical absorption edges. The noted dip in the IPCE plot after 450 nm is correlated to the absorption spectrum and the observed pre-edge in the Tauc plot of the PG2 membrane, Figure 5c.

A tiny outside voltage is applied between the PEC cell’s working and counter electrodes, and the electrical energies injected into the cell must be deducted. This is possible by applying a small external voltage to the PEC cell and calculating the *ABPE* values for the designed electrode utilizing Equation (9) [50]:

(9) $$ABPE\;(\%)=\frac{J_{ph}\;\times \;(1.23-V_{app})}{P}\times 100$$

where 1.23 is the standard state reversible potential of H_2_O and *V_app_* is the externally applied voltage. The values of ABPE vs the applied potential were studied as a function of incident light wavelength, as shown in Figure 7d. It can be seen that PES modified with GO showed a substantial increase in ABPE to reach 7.1% @ −1 V and 390 nm, which is in good agreement with the J_ph_-V curve shown in Figure 6a.

**Figure 8:**
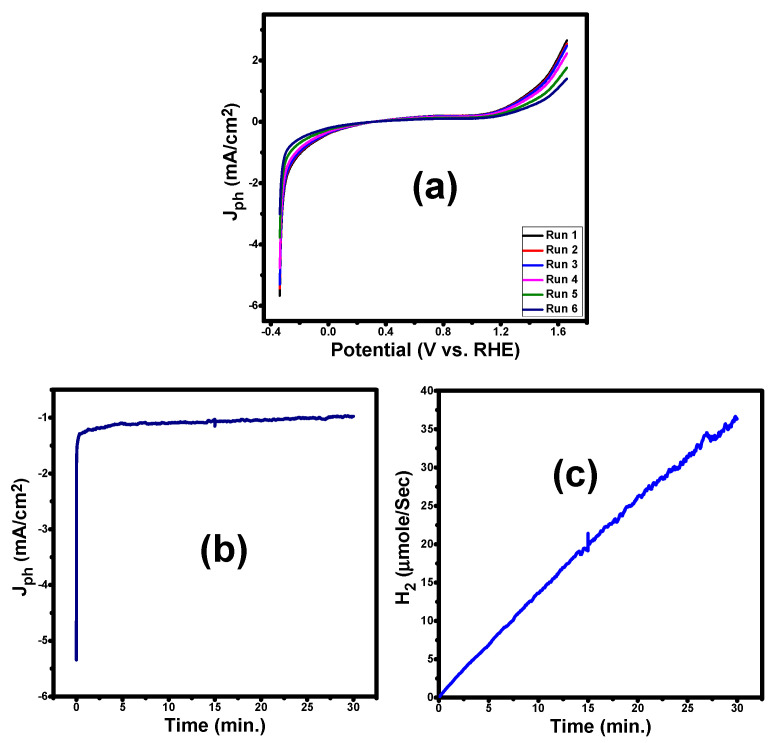
(**a**) The reusability of the PG2 after 6 runs, (**b**) chronoamperometry J_ph_-t response of PG2, and (**c**) the calculated number of H_2_ moles using PG2 membrane.

In general, photoelectrode stability is a critical factor for realistic industrial applications of PES-based photoelectrodes. For evaluating the reusability and stability of the optimized sample, we have measured the J_ph_-V curves as a function of the number of reusability runs and the chronoamperometry J_ph_-t response at −1 V for PG2, Figure 8a,b. From Figure 8a, the current density of the PG2 membrane reserved 95% of its initial efficiency after 6 runs. The reached performance after 6 runs of reusability is very high compared to the previously studied electrodes [55,56]. The membrane performance after six runs of reusability was attributed to the high thermal and mechanical stability and conductivity of the incorporated GO.

The stability of PG2 was also evaluated with a steady-state chronoamperometry by measuring J_ph_-t response at −1 V, which was presented in Figure 8b. The results showed that its photocurrent density rapidly declined within 25 s to reach 1.01 mA/cm^2^, then the current density slightly decreased to reach 0.974 mA/cm^2^ within 30 min as shown in Figure 8b. I.e., the electrode after 30 min preserves 96.4% of its detected photocurrent at 25 s. For more evidence, the J_ph_-t response of the PG2 electrode is studied again for 180 min and the data are presented in Figure S2 (Supplementary Data). The magnitude of the photocurrent density declined to 0.644 mA/cm^2^ after 180 min. This means that the electrode preserved 65.7% of its observed performance at 25 s. According to recent studies [57,58], the three main factors that can result in a reduction in the photocurrent of a photoelectrode that contains an organic photoactive layer over time are a decline in chemical stability, photo corrosion from accumulated surface-charging holes, and a decline in the photostability of the organic photoactive layer itself. Whereas the organic photoactive layer’s low photostability was counted to be the primary cause of the destabilization [59,60]. It indicates that the passivation technique utilizing GO, although beneficial to increase chemical stability and reduce photocorrosion by accumulated surface charges, is still insufficient based on the observed small decrease in photocurrent density over the 180 min operation. As a result, the primary goal of our future work can be to thoroughly investigate electrode stability and implement more practical techniques, such as the inclusion of plasmonic nanoparticles-decorated GO to enhance photostability and decrease surface charge accumulation.

The number of H_2_ moles produced by the PEC cell can be estimated using Equation (10) [32].

(10) $$H_{2}\left(moles\right)=\int_{0}^{t}\frac{J_{ph}}{F}dt$$

Here, *t* is the generation period, and *F* is the Faraday constant (9.65 × 10^4^ C/mol). The variation of *H*_2_ (moles) versus production time is depicted in Figure 8c. The rate of *H*_2_ production is 0.7 mmol h^−1^ cm^−2^.

Typically, under solar illumination, PES acts as an ion conductor and prevents any fuel diffusivity, and GO can act as a light harvester and absorb incident light to generate photoexcited e^−^/h^+^ pairs, which are prone to dissociate at the interface between GO and PES, wherein the photo-excited electrons are injected from GO to PES and collected by substrate [54]. Here, a thin blocking layer (BL) was formed between the PES/GO membrane and substrate for inhibiting charge recombination of back-electrons. Subsequently, as presented in Figure S3 (Supplementary Data), the collected electrons traveled through the external circuit to the Pt counter electrode to generate the H_2_ fuel whereas the electrolyte acts as a hole scavenger to sacrifice the positive holes and increase the performance of H_2_ evolution [61]. Finally, the PEC results attained in our study were compared with many earlier reported photoelectrodes for water splitting as shown in Table 2 [62,63,64,65,66]. The obtained values of J_ph_, IPCE (%), and ABPE (%) confirmed that the PES/GO MMW membrane as a flexible electrode is very efficient and suitable for photoelectrochemical water splitting application under visible light irradiation.

## 5. Conclusions

Here, innovative and inexpensive flexible membranes made from PES and various GO ratios were constructed using the phase inversion approach to determine the best electrode for PEC hydrogen production. These membranes were characterized using different techniques including XRD, FTIR, and SEM. The optimum membrane appears as layers with a random distribution of GO nanosheets which facilitates the movement of electrons into these layers for hydrogen production. The optimized GO ratio in the membrane was estimated to be 0.008 wt.%. The incident photon-to-current efficiency (IPCE) was estimated to be 14.4% at *λ* = 390 nm. Furthermore, this membrane has significant stability after 6 cycles of reusability which was attributed to its high ionic conductivity. Finally, these membranes can be thought of as a brand-new class of flexible electrodes that can be utilized for PEC cell construction and effective hydrogen production.

## Figures and Tables

**Table 1:** XRD parameters for GO nanosheets.

Parameter	GO Nanosheets			
	(001)	(220)	(221)	(322)
Position (2 θ)	10.50°	21.05°	26.73°	42.26°
The crystallite size (nm)	23.17	19.47	40.49	10.19
Texture coefficient (TC)	3.66	0.11	0.18	0.05
d-spacing (Å)	8.423	4.219	3.335	2.139
Dislocation density/nm^2^ (*δ*) × 10^−3^	1.9	2.6	61.1	9.6
*a* = *b* (Å)	11.94			
*c* (Å)	4.33			
*V* (Å^3^)	617.3			

**Table 2:** Comparison of the values of J_ph_, IPCE (%), and ABPE (%) for the present work and earlier studied PEC catalysts based on GO.

Catalyst	J_ph_ (mA/cm^2^)	IPCE (%)	ABPE (%)	Ref.
PES/GO MMW membrane	5.7	14.4	7.1	This Work
Poly(m-toluidine)/rolled graphene oxide nanocomposite (PMT/roll-GO)	0.08	0.92	0.9%	[62]
3D porous g-C_3_N_4_/GO-M (Au, Pd, Pt) composite catalysts	0.9	-	-	[63]
Graphite/rolled graphene oxide/carbon nanotube (G/R-GO/CNT)	1.5	8.4	8	[64]
PbS/graphene oxide/polyaniline	1.9	9.4	6.17	[65]
Decoration of graphene oxide as a cocatalyst on Bi-doped g-C_3_N_4_	0.4	1.6	1.62	[66]

## Data Availability

The data presented in this study are available on request from the corresponding author.

## Supplementary Materials

The following supporting information can be downloaded at: https://www.mdpi.com/article/10.3390/membranes13070653/s1. Table S1. Values of electronic conductance and ionic conductance of PES/GO electrode. Figure S1. I-V characteristics of the PES/GO electrodes for (a) electronic conductance and (b) ionic conductance calculations. Figure S2. chronoamperometry J_ph_-t response of PG2 for 180 min at 0.3 M of sodium sulfate (Na_2_SO_4_) aqueous solution under applied bias voltage of −1 V. Figure S3. Schematic diagram for PEC water splitting using PES/GO electrode.
