# Supplementary material for: Flexible Electrode Based on PES/GO Mixed Matrix Woven Membrane for Efficient Photoelectrochemical Water Splitting Application
Source: Membranes (Basel). 2023 Jul 8;13(7):653. doi: 10.3390/membranes13070653 (PMC10384634; doi:10.3390/membranes13070653)
Supplement: Supplementary file 1 [file membranes-13-00653-s001.zip › membranes-2343942-supplementary.pdf]

Supplementary Data

Flexible electrode based on PES/GO Mixed matrix woven membrane for efficient photoelectrochemical water splitting application

Ghadah M. Al-Senani [1], Mohamed Zayed [2], Mervat Nasr [2,3], Sahar S. Ali [4], Mohamed Shaban [5,2]*, Fatma Mohamed [2,3,6]

[1] Department of Chemistry, College of Science, Princess Nourah bint Abdulrahman University, P.O. Box 84428, Riyadh 11671, Saudi Arabia
[2] Nanophotonics and Applications Lab, Physics Department, Faculty of Science, Beni-Suef University, Beni-Suef 62514, Egypt
[3]Chemistry Department, Faculty of Science, Beni-Suef University, Beni-Suef 62514, Egypt
[4] Chemical Engineering and Pilot-Plant Department, National Research Center, P.O. Box 12622, Dokki, Cairo, Egypt
[5] Department of Physics, Faculty of Science, Islamic University of Madinah, P. O. Box: 170, Madinah 42351, Saudi Arabia
[6] Materials Science Research Laboratory, Chemistry Department, Faculty of Science, Beni-Suef University, Beni-Suef, Egypt
* Correspondence: author: mssfadel@aucegypt.edu

Table S1. Values of electronic conductance and ionic conductance of PES/GO electrode.

Sample	PG0	PG1	PG2	PG3	PG4	PG5
Electronic Conductance (µS)	4.85869E-4 ± 2.28843E-6	0.02429 ± 1.14421E-4	0.09717 ± 4.57686E-4	0.02186 ± 1.02979E-4	0.01992 ± 9.38256E-5	0.01822 ± 8.58161E-5
Ionic Conductance (mS)	0.16223 ± 0.00167	0.70683 ± 0.00182	2.71506 ± 0.00307	0.14521 ± 0.00167	0.12819 ± 0.00166	0.10096 ± 0.00166

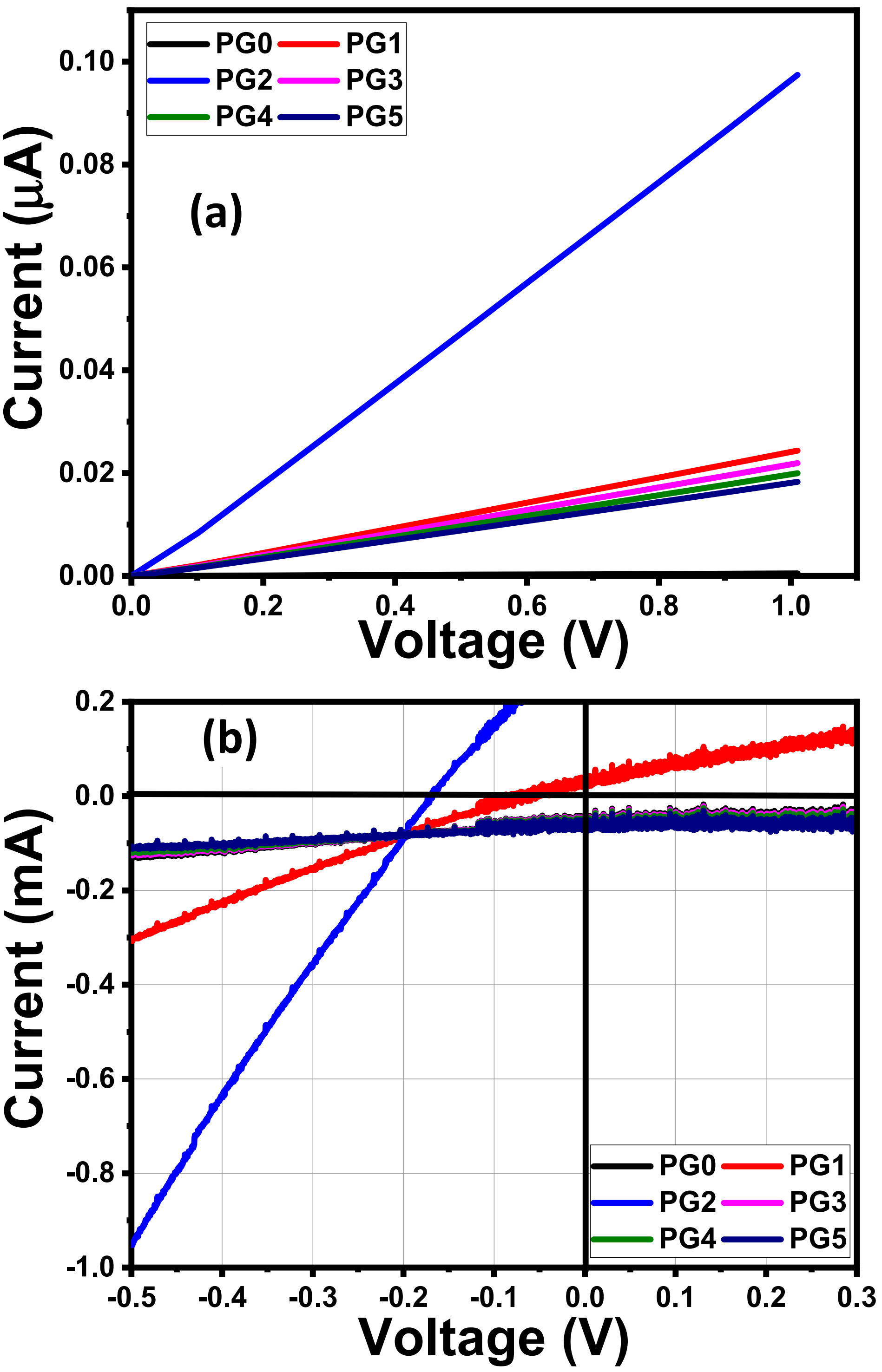

Figure S1. I-V characteristics of the PES/GO electrodes for (a) electronic conductance and (b) ionic conductance calculations.

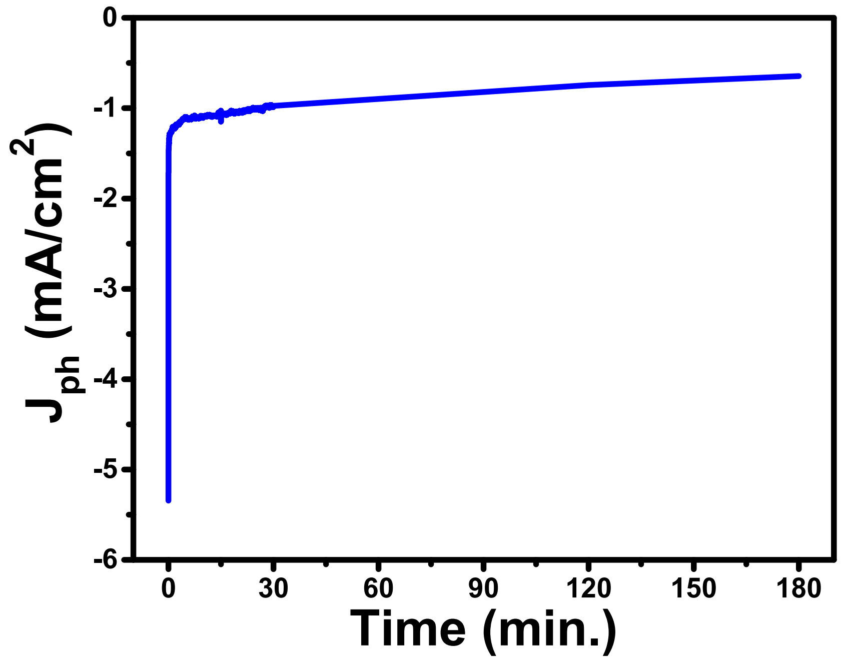

Figure S2. chronoamperometry J_{ph}-t response of PG2 for 180 min at 0.3 M of sodium sulfate (Na_2SO_4) aqueous solution under applied bias voltage of -1V.

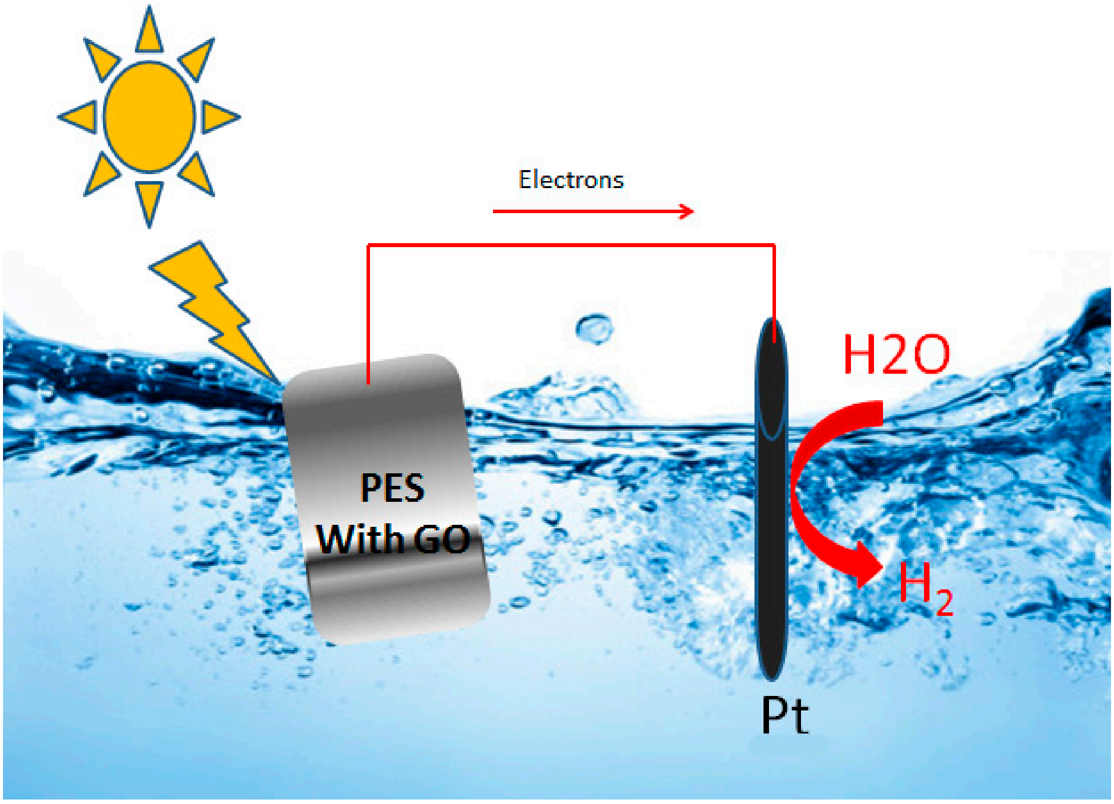

Figure S3. Schematic diagram for PEC water splitting using PES/GO electrode.
